# Ammonium Formate as a Safe, Energy-Dense Electrochemical Fuel Ionic Liquid

**DOI:** 10.1021/acsenergylett.2c01826

**Published:** 2022-09-06

**Authors:** Zachary J Schiffer, Sayandeep Biswas, Karthish Manthiram

**Affiliations:** † Department of Chemical Engineering, Massachusetts Institute of Technology, 77 Massachusetts Avenue, Cambridge, Massachusetts 02139, United States; ‡ California Institute of Technology, 1200 East California Boulevard, Pasadena, California 91125, United States

## Abstract

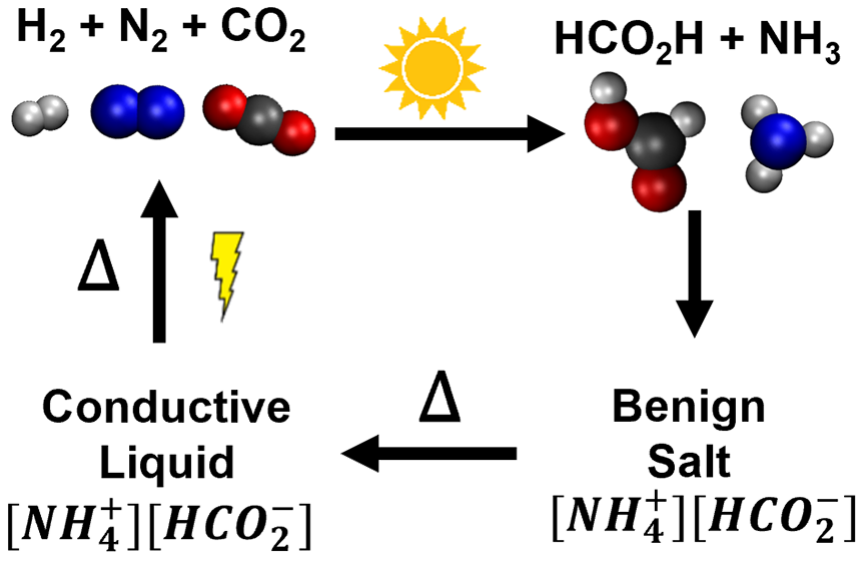

While solid and liquid energy carriers are advantageous due to their high energy density, many do not meet the efficiency requirements to outperform hydrogen. In this work, we investigate ammonium formate as an energy carrier. It can be produced economically via a simple reaction of ammonia and formic acid, and it is safe to transport and store because it is solid under ambient conditions. We demonstrate an electrochemical cell that decomposes ammonium formate at 105 °C, where it is an ionic liquid. Here, hydrogen evolves at the cathode and formate oxidizes at the anode, both with ca. 100% Faradaic efficiency. Under the operating conditions, ammonia evaporates before it can oxidize; a second, modular device such as an ammonia fuel cell or combustion engine is necessary for complete oxidation. Overall, this system represents an alternative class of electrochemical fuel ionic liquids where the electrolyte is majority fuel, and it results in a modular release of hydrogen with potentially zero net-carbon emissions.

As renewable electricity from sources such as solar and wind becomes increasingly available, it is important to develop methods to store and transport this energy. Lithium-ion batteries are a convenient and cheap method for storing and transporting renewable energy, but they are limited by their energy density of 0.2–0.7 kWh/L (Table 1) relative to chemical carriers of renewable electricity. Hydrogen has been investigated widely as a renewable and carbon-free fuel, but large energy losses incurred in compression have limited its use as an energy carrier for clean electrons. Hydrogen gas has a higher energy density than lithium-ion batteries, but this density is lower than those of other liquid fuels; typically it is stored as either a compressed gas at 700 bar and 298 K or liquified at 1 bar and 20 K, with an energy density of 1.3 or 2.3 kWh/L, respectively (Table 1).^1,2^ Neither option is ideal because compressed gas storage requires large volumes and high costs, and liquified hydrogen, although representing a higher hydrogen content per unit volume than compressed hydrogen gas, is energy intensive due to liquification and costly for long-term storage due to boil-off.

**Table 1:** Potential Renewable Energy Carriers

energy carrier	energy density (kWh/L)^a^	conditions	disadvantage
Li-ion battery	0.2–0.7	ambient *P*, *T*	low energy density
H_2_ (liq)	2.3	20 K, 1 bar	transport + storage
H_2_ (gas)	1.3	298 K, 700 bar	low energy density
NH_3_ (liq)	3.3	298 K, 10 bar	safety + energy extraction
formic acid	2.0	ambient *P*, *T*	safety + energy extraction
ammonium formate	3.2	ambient *P*, *T*	energy extraction

a The energy density given is calculated from the Gibbs free energy of reaction to complete combustion products at 25 °C (N_2_, CO_2_, H_2_O(l)). Using the enthalpy of reaction instead has little effect on the trends in calculated energy density. Details of calculations and enthalpy values are provided in Supporting Analysis.

For these reasons, alternative solid and liquid carriers of renewable energy have been investigated; here we briefly consider ammonia and formic acid, two well-studied energy carriers, but there are many potentially promising options. From the perspective of energy density, formic acid is appealing since it is a liquid under ambient conditions, while ammonia has the disadvantage that its liquification requires modest cooling to −33 °C or pressurizing to 10 bar.^1^ Formic acid, however, has a lower energy density of 2.0 kWh/L,^3^ less than that of pressurized liquid ammonia, 3.3 kWh/L (Table 1).^1^ Both of these carriers present safety challenges that restrict their deployment, as they are corrosive, making handling difficult. Ammonia is also concerning in the context of accidental release, since it is toxic at concentrations greater than 5000 ppm.^4^ Despite these disadvantages, both energy carriers are produced at scale and already have existing infrastructure for storage and transportation. In addition, both of these carriers have the potential to be produced using renewable electricity and air; formic acid could be made from carbon dioxide reduction,^3,5,6^ and ammonia could be produced from either the Haber–Bosch process using renewable electricity and hydrogen or electrochemical nitrogen reduction.^7−11^ While these energy carriers have a lot of potential, one of the main problems preventing the uptake of ammonia and formic acid as renewable energy carriers is the extraction of energy from them. Formic acid fuel cells remain inefficient and can suffer from catalyst poisoning.^6,12−19^ Ammonia fuel cells similarly require large overpotentials and suffer from catalyst poisoning.^10,20−22^ Thermal methods for ammonia cracking rely on high temperatures and make the most economic sense at large scales.

To begin to address the aforementioned problems, we focus on an alternative class of energy carrier: electrochemical fuel ionic liquids (EFILs). Ionic liquids and moderate-temperature molten salts have been widely explored as propellants.^23^ The low vapor pressure and high thermal stability of many ionic liquids, such as hydroxylammonium nitrate, have enabled applications as safer, low-toxicity alternatives to existing propellants, such as hydrazine. Ionic liquids are appealing candidates as electrochemical fuels, since they themselves are ionic conductors, a critical property of the electrolyte in an electrochemical device.^24^ Ionic liquids are intrinsically compelling as fuels for the same reasons that ionic liquids have emerged as safe alternatives to conventional propellants: their low vapor pressures and thermal stability greatly enhance safety. This is especially critical in military applications, given the rigorous safety standards that fuels must meet for use in combat environments.^25^ In previous research, ionic liquids have been extensively used as electrolytes in electrochemical devices for many of the same reasons stated above.^26−31^ For example, ionic liquids have been used in fuel cell applications, and oxygen reduction in a wide variety of protic ionic liquids has previously been studied.^29,32^ Ionic liquids are therefore extremely compelling as electrochemical fuels since they are ionically conductive, serving a dual purpose as a fuel and an electrolyte.

Perhaps the simplest EFIL we could envision is prepared by combining the two energy carriers mentioned above, ammonia and formic acid, to generate ammonium formate. For a new candidate fuel, one should consider the routes by which it would be synthesized (reductive chemistry) and how it would be transported and stored, as well as how it would be utilized (oxidative chemistry). In an energy paradigm based on ammonium formate (Figure 1a), both ammonia and formic acid are synthesized via renewable routes. Ammonium formate is then produced by the simple acid–base reaction of ammonia and formic acid; the product has an energy density of 3.2 kWh/L, on par with that of liquid ammonia and higher than those of formic acid and hydrogen (Table 1). The transport and storage of ammonium formate are similarly simple and safe, since it is a benign solid under ambient conditions. This is a significant advantage compared to, e.g., hydrogen storage, where compression is 75% of the combined cost of the compression, storage, and dispensing of hydrogen.^33^ Thus, the remaining question is how to extract the energy from ammonium formate, since the extraction efficiency must be high enough to make the overall energy balance favorable compared to hydrogen. Previous studies have investigated ammonium formate decomposition in aqueous environments^15,34−36^ as well as the decomposition of ammonium formate–formic acid mixtures.^37−41^ These studies are generally limited to a saturated solution of ammonium formate, not a solution that is primarily ammonium formate, and they do not tend to use both temperature and voltage to drive decomposition. Additionally, previous studies have investigated the thermal decomposition of ammonium formate at elevated temperatures for selective catalytic reduction (SCR) applications (>160 °C).^42,43^

**Figure 1:**
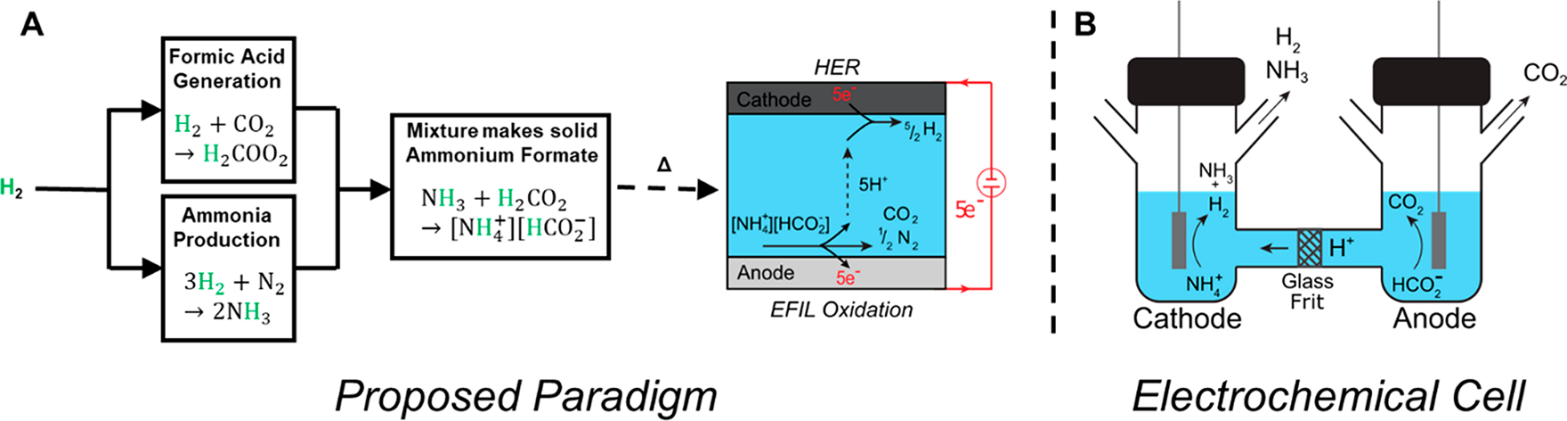
Proposed paradigm for hydrogen storage with ammonium formate (A) and schematic of an experimental H-cell (B). As seen, green hydrogen can be fed into traditional or sustainable plants for formic acid and ammonia generation, and then ammonia and formic acid will spontaneously combine to form ammonium formate. Ammonium formate can be fed into an electrochemical cell, where an applied potential can decompose it to hydrogen, nitrogen, and carbon dioxide. In practice, our experimental cell reduces ammonium to hydrogen at the cathode and oxidizes formate to carbon dioxide at the anode.

We propose that by leveraging the fact that ammonium formate melts at ∼120 °C, forming a conductive liquid, it can be decomposed electrochemically into benign gases while extracting work. Unlike previous studies that focus on either thermal decomposition or low-temperature electrochemical oxidation of <50 w/w% ammonium formate, we utilize both temperature and voltage to decompose molten ammonium formate in an electrolyte with at least 90 w/w% ammonium formate. A complete comparison of our work to previous literature reveals how, through experiments investigating the influence of temperature, anode catalyst, time, and system thermodynamics of ammonium formate as an energy carrier, this work expands on previous studies of ammonium formate and formic acid (a full comparison is given in a Supporting Discussion in the Supporting Information). In addition, previous studies on thermochemical ammonium formate decomposition achieve reaction rates that are significantly lower than the electrochemical reaction rates in this study (a full comparison is given in the Supporting Discussion in the Supporting Information). With electrochemical decomposition, ammonium formate could theoretically be directly decomposed to water, carbon dioxide, and nitrogen in the presence of oxygen in a fuel cell. Alternatively, ammonium formate could be decomposed into another fuel (e.g., hydrogen) which can then be used in a second, modular device to extract work (e.g., in a fuel cell or combustion engine).

In this work, we find that a small, applied potential can decompose ammonium formate into carbon dioxide, hydrogen, and ammonia using gold, palladium, and platinum electrocatalysts as anodes (Figure 1b). In principle, the cathodic gas output of hydrogen and ammonia could be fed either into a secondary fuel cell for electrical work extraction or into a combustion engine for mechanical work extraction. We examine the energy landscape of ammonium formate decomposition and show that the small energy inputs required to melt the system or extract the hydrogen content are negligible compared to the energy content of the system. Overall, in this work we establish EFILs as an alternative class of fuels and demonstrate how ammonium formate, a prototypical example of this class, can be decomposed electrochemically to extract energy, representing how this class of fuel has the potential to store and transport renewable energy effectively.

In an electrochemical cell (Figure 1b), the ammonium cations can be reduced at the cathode to hydrogen gas and the formate can be oxidized at the anode to carbon dioxide. As catalysts for these reactions involving molten ammonium formate are not well-established, we chose to investigate palladium-, platinum-, and gold-foil anodes (Figure 2; the gold electrode assembly is shown in Figure S1) while using platinum as the cathode, since it is known to be active for hydrogen evolution. These initial candidate metals were chosen for their simplicity compared to more complex materials and their general stability as anode materials. From linear scan voltammetry (LSV) (Figure 2a) as well as chronopotentiometry (Figure 2b), we find that palladium anodes were the most active, followed by gold; platinum was the least active. We also find that, over the course of 60 min, the potential remains steady at an applied current of 10 mA (Figure 2b). Slight increases in potential over 60 min are likely due to changes in solution resistance (further discussed in the Supporting Analysis and Figure S2 in the Supporting Information). Note that the system we studied contained 10 w/w% water so that the system formed a liquid at the operating temperature of 105 °C (Figure S6).

**Figure 2:**
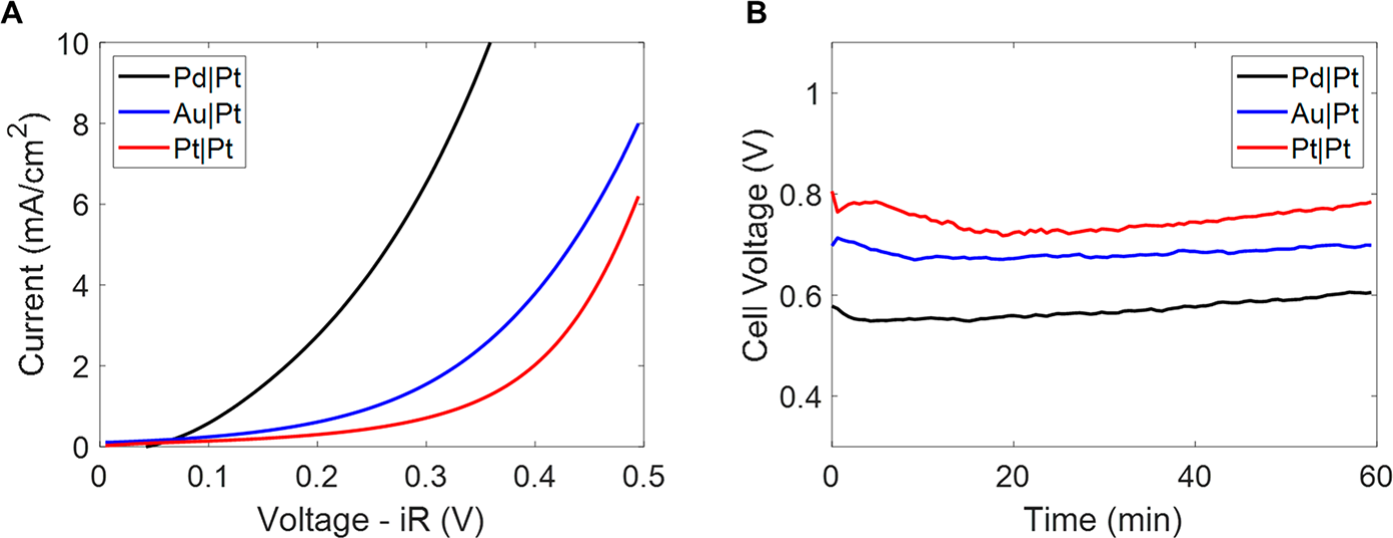
Linear sweep voltammetry (LSV) of ammonium formate with various anode materials (A) and chronopotentiometry with the same setups for 60 min at 10 mA applied current (B). LSVs were performed at a 10 mV/s scan rate. Both experiments consisted of an ammonium formate electrolyte composed of 10 w/w% water at 105 °C. The system is *iR* compensated at 85% for the LSVs so that the kinetic overpotentials as a function of metal can be compared. *iR* compensation is not possible for the chronopotentiometry, as the resistance increases slightly over time, likely due to small changes in electrolyte composition during operation. Cells are defined as “Anode|Cathode”.

We found that, although pure ammonium formate can be used as a liquid at 120 °C, there is a background thermal decomposition that produces small, but measurable, amounts of carbon dioxide, ammonia, and carbon monoxide. Not only does carbon monoxide represents an operational safety concern but also trace amounts of carbon monoxide can poison catalyst surfaces, leading to inefficient catalysts and unstable voltages. At 105 °C, there is no thermal decomposition of ammonium formate according to a gas chromatographic analysis; however, there is some evaporation of ammonia from the system (quantified in the Supporting Analysis in the Supporting Information). Thus, we included some water in this system to lower the operating temperature into a region where thermal decomposition to carbon monoxide is negligible.

There is an inconsistency between the voltages shown in the LSV experiments (Figure 2a) and the chronopotentiometry experiments (Figure 2b) because we used resistance compensation in the LSV experiments. Due to the unoptimized geometry of our H-cell (∼6 cm between electrodes), there was generally ∼25 Ω of resistance in the cell (at 10 mA, this corresponds to an extra 250 mV of potential). This resistance is an important consideration from an energy balance perspective, but it does not affect our analysis, and future cell optimization will reduce this significantly.

Using a platinum cathode and palladium anode, we investigated the Faradaic efficiency of this system, quantifying hydrogen and nitrogen via gas chromatography, carbon dioxide via reactive capture to form CaCO_3_ (Supporting Experimental Procedure and Supporting Analysis, Figures S3 and S4, in the Supporting Information), and ammonia via a colorimetric assay (Supporting Experimental Procedures in the Supporting Information) (Figure 3a). First, nitrogen was not found as a product; this is expected, since ammonia oxidation is a kinetically challenging electrochemical reaction, particularly in this system (discussed further below). At the cathode, we found that the average instantaneous Faradaic efficiency toward hydrogen gas throughout 60 min of operation is 106 ± 2% (Figure 3b). At the anode, we quantified the total amount of carbon dioxide gas cumulatively produced over 60 min and found that the Faradaic efficiency toward carbon dioxide is 98 ± 4% (Figure 3b). There is evidence of ammonia in an acid trap postcell, but the amount of ammonia far exceeded the expected stoichiometric amount associated with formic acid oxidation, indicating that under these operating conditions there is some evaporation of ammonia from the ionic liquid, leaving formic acid behind (Supporting Analysis, Figure S7, in the Supporting Information). We expect that, in our current system, there is an equilibrium between the ammonium formate and gaseous ammonia (eq 1).
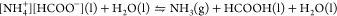
1This equilibrium combined with a flow of carrier gas results in excess ammonia in the postcell trap (quantified in Figure S7). From all the data, we can conclude that the ammonium is being reduced at the cathode to hydrogen gas and ammonia, which *both* leave the cell as gases (eq 2).
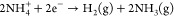
2At the anode, formate is oxidized to carbon dioxide, with additional formate ions likely acting as proton acceptors (eq 3), given that ammonia, which would otherwise be a more competent proton acceptor, is generated far away at the cathode and tends to volatilize.
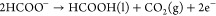
3Small amounts of formic acid (boiling point 100.8 °C) generated at the anode can evaporate in the absence of a more competent proton acceptor such as ammonia. Sufficiently fast transport and equilibration between the anode and cathode could mitigate both ammonia and formic acid evaporation by generating ammonium cations and formate anions, which are less volatile than ammonia and formic acid. Ultimately, the formic acid will be available at the cathode for proton reduction so that the net overall electrochemical reaction (in addition to the slight thermal evaporation of ammonia) is simply the decomposition of one formula unit of ammonium formate (eq 4).
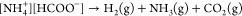
4

**Figure 3:**
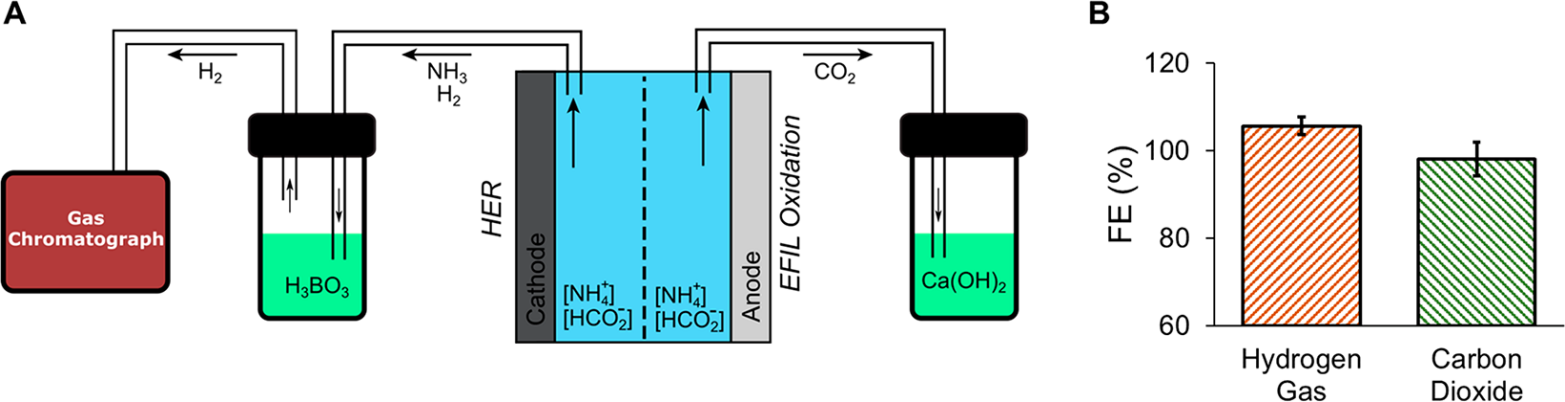
Schematic of experimental setup for quantifying FE (A) and FE closure for hydrogen and carbon dioxide over a 60 min applied current of 10 mA (B). Note that in practice the cell was an H-cell with a glass frit (Figure 1b) and this schematic is a simplification of the actual setup (see Supporting Experimental Procedures in the Supporting Information for details). The hydrogen gas represents an average throughout the 60 min experiment. The carbon dioxide represents the average of two trials where the total mass of carbon dioxide produced during the 60 min experiment was quantified.

While ideally the ammonia would be oxidized at the anode along with the formate, in practice ammonium cation oxidation is extremely difficult; even neutral ammonia oxidation in nonaqueous media is an outer-sphere reaction with >1 V overpotential.^44^ Any ammonia in the system evaporates quickly under the operating conditions. An improved cell design and modification with additives could enable ammonia oxidation, but even in its current form, this ammonium formate electrochemical device has practical utility. For example, the output stream of ammonia can be fed into an ammonia fuel cell. Another solution would be to take advantage of the cathodic output, namely ammonia and hydrogen gas, and feed it to an internal combustion engine to extract mechanical work.

Current research on ammonia electro-oxidation has found the low efficiencies difficult to overcome, but there is a significant amount of ongoing work on using ammonia in combustion engines.^45−55^ One of the main problems hindering ammonia combustion engines is the high autoignition temperature of ammonia (923 K),^52^ which is too high for small-scale combustion engines but can potentially be overcome in larger applications such as cargo shipping. One of the solutions proposed for small-scale combustion of ammonia is to include a secondary fuel for facile combustion and smooth operation, but such a strategy is limited because a user would need to carry two fuels instead of one, in addition to the problems posed by safely storing and transporting ammonia.^45−48^ However, this system’s output stream contains a mixture of ammonia and hydrogen. The cathodic output is therefore a combustible stream that can be fed into an ammonia combustion engine for efficient work extraction.^51,53^ Hence, ammonium formate as presented here, with a small input of electrical energy, could enable dual-fuel ammonia combustion engines.

So far, we have discussed practical methods for energy extraction from ammonium formate. However, knowledge of the theoretical bounds limiting energy extraction is necessary. In general, there are two metrics for quantifying the energy density of a fuel. The first is the enthalpy of reaction—this is the method commonly used and leads to well-known quantities such as the lower heating value or the higher heating value. A second metric would be to use the Gibbs free energy of reaction—this quantity is related to the equilibrium potential of the reaction and thus a metric of the fuel cell’s open circuit voltage. In this system, there is the additional fact that ammonium formate melts at an elevated temperature. The enthalpy and Gibbs free energy of most common gases are well-documented (in this case, ammonia, nitrogen, oxygen, hydrogen, water, and carbon dioxide). The enthalpy and Gibbs free energy for solids and ionic liquids, however, are not always accessible. In the case of ammonium formate, the solid enthalpy of formation has been tabulated (−567.5 kJ/mol),^56^ but the entropy and Gibbs free energy have not. Instead, volume-based methods can be used to calculate the entropy of the ammonium formate solid.^57−59^ This method has been found to be widely applicable to a range of ionic solids with an error of less than ∼10%.^58^ Using this method, the entropy of ammonium formate can be determined to be 126.2 J/(mol K) (Supporting Analysis, Table S2, in the Supporting Information). Using differential scanning calorimetry, we measured that the melting point of ammonium formate is 120 °C with an enthalpy of fusion of 43.2 kJ/mol (Supporting Analysis, Figure S5, in the Supporting Information). From these values, the entropy of fusion was calculated to be 109.9 J/(mol K). Assuming that the enthalpy and entropy of fusion are not functions of temperature, the thermodynamic landscape under ambient conditions and at 120 °C (the melting temperature) can be analyzed (Figure 4). Specifically, solid ammonium formate can be turned into a liquid with a small input of energy given by the enthalpy of fusion.

**Figure 4:**
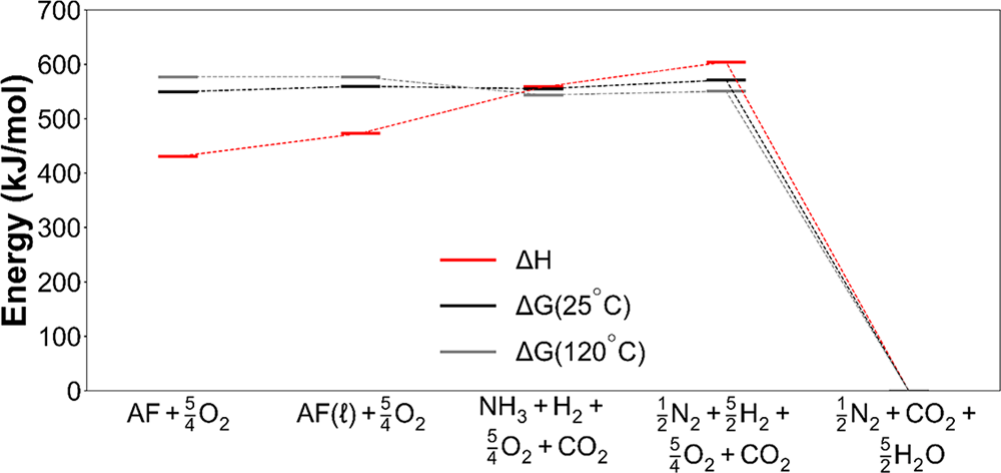
Thermodynamic landscape of ammonium formate decomposition reaction (detailed calculations are given in the Supporting Analysis in the Supporting Information). The enthalpy and Gibbs free energy of decomposition to nitrogen, water (gas), and carbon dioxide of ammonium formate and possible intermediate mixtures are shown. The Gibbs free energy at operating temperatures does not differ significantly from its values under ambient conditions. Detailed calculations are given in the Supporting Analysis in the Supporting Information.

Then, with very little theoretical work, liquid ammonium formate can be decomposed into either ammonia, hydrogen, and carbon dioxide or nitrogen, hydrogen, and carbon dioxide. Importantly, either decomposition requires only a small amount of work and therefore the resulting mixture retains its energy content as a fuel. While the Gibbs free energy of decomposition remains roughly constant as hydrogen is extracted (Figure 4), the enthalpy of decomposition increases—this balances the entropy as the number of gas molecules increases.

In terms of electrochemical potential, *E*_eq_ for the conversion of solid ammonium formate under ambient conditions to hydrogen, carbon dioxide, and nitrogen is −45 mV and *E*_eq_ for the conversion of liquid ammonium formate at 120 °C is +55 mV; these values are small compared to the 1.23 V of a hydrogen fuel cell. Decomposition to hydrogen, carbon dioxide, and ammonia (not nitrogen gas) has an *E*_eq_ value of −11 mV starting from solid ammonium formate at 25 °C and +69 mV starting from liquid ammonium formate at 120 °C (detailed calculations are given in the Supporting Analysis in the Supporting Information); this further suggests that the main source of energy is from oxidizing hydrogen to water, and the various small energy exchanges required to get solid ammonium formate to hydrogen gas are negligible compared to the final oxidation of hydrogen.

With theoretical equilibrium potentials, we can calculate kinetic overpotentials for our system. As mentioned previously, the nonideal cell geometry results in significant resistance (∼25 Ω). Using a palladium anode, the LSV reveals that the resistance-compensated cell voltage required to drive 10 mA/cm^2^ is ca. −360 mV. This corresponds to a ca. 420 mV overpotential relative to the equilibrium potential of liquid ammonium formate decomposing into hydrogen, ammonia, and carbon dioxide at 105 °C, as the equilibrium potential is +57 mV at this temperature. Ignoring the energy input required to heat and melt the system, the kinetic overpotential relative to the equilibrium potential of *solid* ammonium formate decomposing into hydrogen, ammonia, and carbon dioxide starting at 25 °C is ca. 350 mV (the equilibrium potential being −11 mV). These overpotentials are very reasonable for a proof-of-concept system using a planar electrode and unoptimized geometry.

In addition to thermodynamic limits and full-cell analyses, the anodic overpotential is extremely important for both understanding the effectiveness of an anode catalyst as well as comparing different systems. While in room temperature aqueous systems standard references such as Ag/AgCl can be used to calculate half-reaction overpotentials, in nonaqueous systems at elevated temperatures, standard reference reactions are not as accessible. Instead, pseudoreferences such as a Pt wire can aid in experimentally probing overpotentials in a consistent manner. Pt pseudoreferences have been successfully used previously for similar ammonium formate–formic acid systems.^39^ We find that with ammonium formate containing 10 w/w% water at 105 °C, a palladium anode oxidizes formate with 10 mA/cm^2^ at less than 0.3 V vs Pt (Figure 5). To put this number in context, formate oxidation at 80 °C and room temperature (22 °C) are compared (Figure 5). These experiments were conducted with added water at each temperature to achieve the desired melting point while retaining as much concentrated ammonium formate as possible; specifically, the electrolyte contained ammonium formate with 25 w/w% water (just under saturation at that temperature) at 80 °C and saturated in water at 22 °C. Unsurprisingly, as the temperature increases, the overpotential required to oxidize ammonium formate decreases relative to a Pt pseudoreference. At 22 °C and 80 °C, the ferricyanide/ferrocyanide redox couple can be used to calibrate the Pt pseudoreference (at 105 °C, the ferricyanide thermochemically decomposes and cannot be used). From ferricyanide redox calibrations, we find that the Pt pseudoreferences are −0.04 and −0.15 V vs SHE at 22 °C and 80 °C, respectively (Table S6). Additionally, the Pt pseudoreference is 0.19 V vs the formate oxidation reaction (eq 2) and 0.05 V vs formate oxidation at 22 °C and 80 °C, respectively (Table S6). This trend combined with the experimentally observed LSVs at multiple temperatures demonstrates that, as the temperature increases, the overpotential relative to the formate oxidation reaction will decrease for this system. A simple mathematical analysis of the influence of temperature on overpotential also supports this conclusion (see the Supporting Discussion in the Supporting Information).

**Figure 5:**
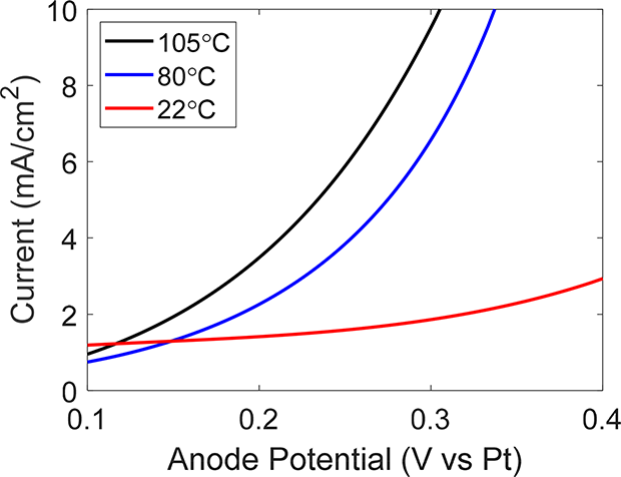
LSVs of the anodic reaction (formate oxidation) at various temperatures at a palladium anode. A Pt pseudoreference was used in all cases. The electrolyte composition was ammonium formate in water at 90 and 75 w/w% and saturation for the 105 °C, 80 °C, and 22 °C systems, respectively. These electrolyte compositions were chosen to maximize ammonium formate concentration while the melting temperature was maintained. All LSVs were 85% *iR*-compensated.

These results match kinetic intuition, namely that temperature increases reaction rates; this principle in electrochemistry is well-established and practical water electrolyzers and fuel cells are often operated at slightly elevated temperatures. Severely elevated temperatures will lead to decreased energy efficiency due to heat loss and thermal decomposition to undesirable side products, but slightly elevated temperatures are both efficient and helpful for improving system kinetics. For example, the enthalpy of fusion for ammonium formate is 43.2 kJ/mol; this corresponds to 90 mV of extra voltage for the five-electron process of converting ammonium formate to nitrogen, hydrogen, and carbon dioxide. This is small compared to the hundreds of millivolts in overpotential saved kinetically by increasing the temperature (Figure 5), justifying the use of slightly elevated temperatures. Additionally, the equilibrium potential of the reaction increases by 85 mV (requiring less input work) when the temperature is increased from 25 to 105 °C, almost completely balancing the enthalpy of fusion without accounting for improved kinetic overpotentials.

Safe, cheap, and energy-dense renewable fuels are essential for renewable energy to replace fossil fuels; however, many proposed fuels do not meet the energy efficiency requirements to outperform hydrogen. In this work, we establish ammonium formate, a combination of ammonia and formic acid, as an energy carrier. Its production is strictly the combination of ammonia and formic acid, which both are produced efficiently at scale, and it is a solid under ambient conditions, making it safe to transport and store. Using an electrochemical cell, we show that an applied potential can decompose ammonium formate into ammonia, carbon dioxide, and hydrogen. Relative to solid ammonium formate at 105 °C, the overpotential of this system is ca. 350 mV with a platinum cathode and a palladium anode; the system is strongly electrode dependent, suggesting a route for further decreasing the overpotential. Additionally, we demonstrate the benefits of temperature on this system as a tool for reducing kinetic overpotentials at the anode. While ammonia can theoretically be oxidized at the anode, in practice we found that ammonia evaporates under these operating conditions. Future electrolyte and system engineering can enable better gas and ion transport, which would prevent gaseous ammonia from escaping. In its current form, our system would require a secondary modular unit, such as an ammonia fuel cell or an internal combustion engine. Overall, this proof-of-concept system is an example of an alternative class of electrochemical fuel ionic liquids where the electrolyte is the fuel, and these experiments and calculations suggest how ammonium formate can modularly store and transport energy with potentially zero net carbon emissions.

## Supporting Information Available

Methods and materials, calculations, additional analyses, and additional discussions (PDF)
