# Supplementary material for: Ammonium Formate as a Safe, Energy-Dense Electrochemical Fuel Ionic Liquid
Source: ACS Energy Lett. 2022 Sep 6;7(10):3260–7. doi: 10.1021/acsenergylett.2c01826 (PMC9578050; doi:10.1021/acsenergylett.2c01826)
Supplement: Supplementary file 1 — nz2c01826_si_001.pdf [file nz2c01826_si_001.pdf]

Supporting Information for Ammonium Formate as a Safe, Energy-Dense Electrochemical Fuel Ionic Liquid

Zachary J Schiffer[1,2†], Sayandeep Biswas[1†], Karthish Manthiram[2,*]

[1]Department of Chemical Engineering, Massachusetts Institute of Technology, 77 Massachusetts Avenue, Cambridge, MA 02139 [2] California Institute of Technology, 1200 E California Blvd, Pasadena, CA 91125

† These authors contributed equally

[*]Corresponding Author: karthish@caltech.edu

Supporting Experimental Procedures 3
Materials 3
Electrochemical experiment preparation 3
Cell preparation 3
Electrode preparation 4
Electrochemical experiments 6
Product Quantification 6
Differential Scanning Calorimeter 8
COSMO-RS Calculations 9
Supporting Discussions 10
Comparison to Previous Literature 10
Comparison to Thermochemical Decomposition 11
Influence of Temperature on Electrochemical Kinetics 12
Supporting Analysis 15
Energy Density Calculations 16
Resistance of cell over time 18
Hydrogen quantification 19

Carbon dioxide quantification 21
Thermodynamic landscape 22
Equilibrium potential calculations 23
Differential Scanning Calorimeter 24
Control Experiments 26
Temperature Dependence Referenced Experiments 26
Temperature Dependence on System Equilibrium Composition 27
Supporting references 29

Supporting Experimental Procedures

Materials

Anhydrous ammonia gas (Airgas, AM AH80N705). Platinum foil, 0.025mm thick, 99.99% trace metals basis (Beantown Chemical, 213815). Gold foil, 0.127mm thick, Premion 99.99% trace metals basis (Alfa Aesar, 00132). Palladium foil, 0.1mm thick, 99.9% trace metals basis (Beantown Chemical, 135850). Nitric acid, TraceMetal grade (Fisher Chemical, A509). Argon UHP grade 5.0 gas (Airgas, AR UHP300). Ammonium Formate >98% (Alfa Aesar, AA14517-30, Lot U27F063). Aluminum Foil Electrical Tape (McMaster-Carr, 76925A103). Aluminum rod, 1/8” diam. (McMaster-Carr, 8974K192).

Electrochemical experiment preparation

Cell preparation

Ammonium formate is dried in a beaker at 100°C under an ammonia atmosphere for at least 4 hours (in practice, the beaker was covered in parafilm, keeping the parafilm away from the heat, and anhydrous ammonia gas was flowed at 50 SCCM into the beaker). Less than 30 g of ammonium formate was dried at a time to ensure sufficient drying. The dried ammonium formate was then stored in a desiccator and used within a few days.

The custom H cell used in these experiments was designed to look like Figure 1b. Namely, the H cell had a glass frit separator and each compartment had two additional openings on the side of gas inlet and outlet. Before each experiment, the outside of the glass H Cell was wiped down with hexanes to remove the silicone oil. The inside of the cell was then thoroughly rinsed with MilliQ water, followed by 10% nitric acid, followed by MilliQ water, followed by acetone, followed by MilliQ water. Then, the cell was dried at 80 °C to remove all water.

After removing the cell from the drying oven, each compartment of the glass cell was filled with 5.4 g of dried ammonium formate. 0.6 g of MillQ water was then added to each compartment. The main opening at the top of the cell compartments were parafilmed closed, but the gas ports were left open for air flow. The cell was then placed in an oil bath at 105°C for 30 minutes to melt the ammonium formate-water mixture. The electrodes were then inserted and the electrochemical experiments conducted. The electrode area in the electrolyte was approximately 1.5±0.1 cm^2.

Electrode preparation

For metal foil cathodes, the foils were polished using 1500 grit sand paper for >1 minutes, sonicated for at least 2 minutes in DI water, and then dried at 80 °C to remove all water. This was done before every experiment unless specified otherwise. Due to the thinness of Pt foils, these foils were not polished with sand paper. Instead, they were dipped in 10% nitric acid for at least a minute (a few hours if a new Pt foil) and then sonicated in DI water and dried as described above.

The electrodes themselves were attached to a small aluminum current collector with Kapton tape, and the aluminum current collector was attached to an aluminum rod with aluminum tape. The potentiostat was then connected to the aluminum rods via alligator clips. Note that contact resistance can be a problem, and lots of tape and finger-tight applied pressure is useful for assuring low resistance between the potentiostat and the electrode foil (**Figure S1**). The electrode area in the electrolyte was approximately 1.5±0.1 cm^2.

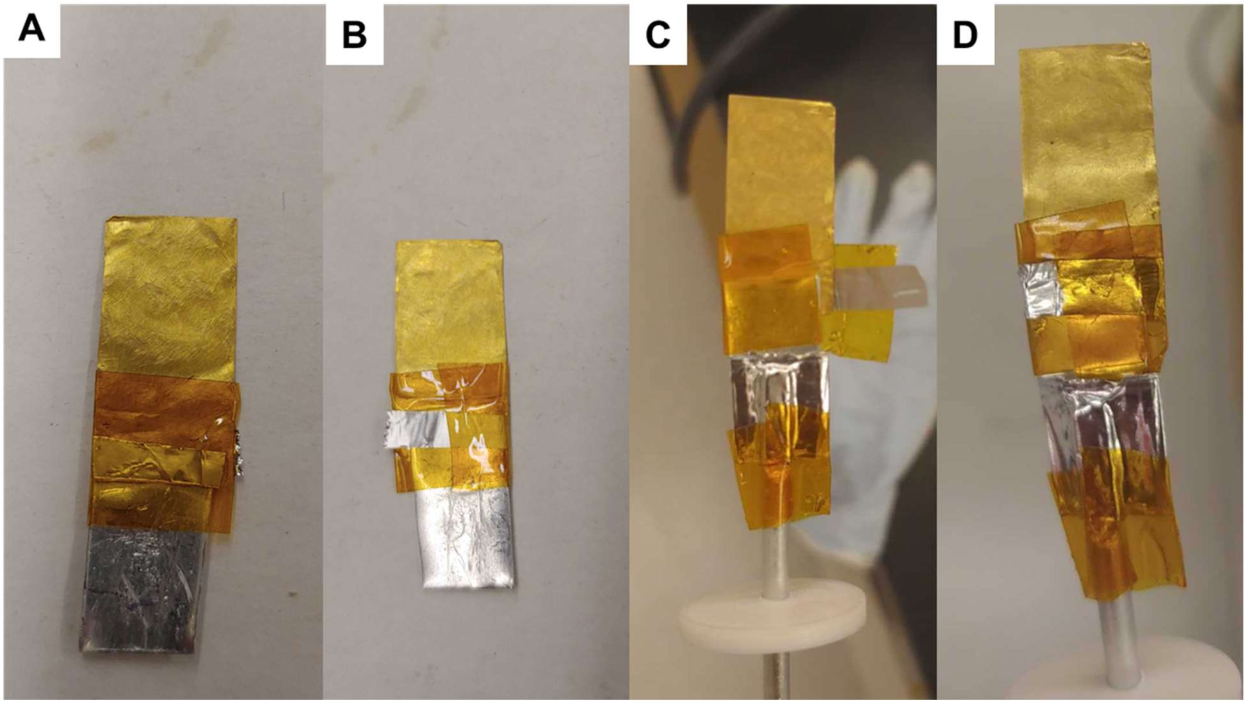

Figure S1. Example electrode preparation for gold electrode. First, the electrode is taped with Kapton tape to an aluminum current collector (A,B). A strip of aluminum foil is often used to help improve electrical contact as well as to create a "tab" (A, B) to help remove the tape after the experiments (a so-called "babybel®" tab). Then, aluminum conductive tape is used to attach

the current collector to an aluminum rod to which the alligator clips will attach (C,D). Additional Kapton tape is applied as necessary to guarantee good electrical contact.

Electrochemical experiments

All electrochemical experiments were performed using a Biologic VMP3 potentiostat. A typical experiment proceeds as follows:

1) A PEIS test to determine the initial impedance, followed by a manual resistance compensation at 85%
2) An LSV at 10 mV/s from 0 V to 0.5 V
3) An open circuit rest for two minutes to add a stir bar and let equilibrate
4) A subsequent PEIS to record any changes in the resistance
5) A chronopotentiometry step with 10 mA applied current for 60 minutes
6) A final PEIS step to note any changes in resistance during the previous step

Product Quantification

Hydrogen quantification was done using an SRI gas chromatograph (GC). A thermal conductivity detector with nitrogen gas as the carrier was used to detect hydrogen gas. Reaction conditions are the same as in other experiments, namely dried ammonium formate combined with 10 w/w% water melted at 105°C. Nitrogen gas was flowed at 5 standard cubic centimeters per minute (SCCM) into the headspace of each compartment. For quantification of hydrogen, a post trap of 0.1 M aqueous boric acid (H_3BO_3) was used to catch any ammonia before the gas flow

entered the GC. The GC was calibrated in the range of interest to quantify hydrogen gas partial currents and Faradaic efficiencies (**Figure S3**). For quantification of carbon dioxide, quantification via GC was not possible due to the necessity of a post trap for the ammonia. The CO2 would dissolve in the post trap, leading to inaccurate GC readings. Instead, a calcium hydroxide trap was used. An empty 20 mL vial was used to collect any possible liquids (sometimes small quantities of electrolyte can travel over) that may be carried along with the gas. The outlet gas from this empty vial was bubbled through a CO_2 trap.

The trap is prepared by dissolving calcium hydroxide in water. When CO_2 is bubbled, it reacts to form calcium carbonate (an insoluble solid) which can later be separated and weighed to calculate the amount of CO_2 captured. The experiments performed to quantify CO_2 included a chronopotentiometry run at 7.1 mA for 1 hour. Through trivial mole balance calculations, it is expected to form 13.24 mg of calcium carbonate and requiring roughly 9.8 mg of calcium hydroxide. At room temperature the solubility of calcium hydroxide in water is 1.6 mg/ml, therefore for a 10 mL trap the maximum dissolved amount is 16 mg. This posed a risk as the concentration of dissolved calcium hydroxide would be reduce as experiment progressed, possibly making the trap less effective. Therefore, a total of 32 mg (double saturation amount) of calcium hydroxide was added to 10 mL of water to prepare the trap.

The extraction of calcium carbonate from the trap must be performed with great caution as the expected yield is small and losses must be minimized. Additionally, the excess calcium hydroxide must be carefully washed off else it will contaminate the final extracted product and overestimate the amount of CO_2 in the trap. The process for extraction is detailed as follows:

1. Transfer the contents of the trap to a 15 mL Falcon tube
2. Centrifuge at 6000 rpm for 10 minutes and remove the water – It was better to leave around 2 mL of water and use a pipette to prevent loss of solids.
3. Add 10 mL of fresh water to the trap container to wash it for any residual and transfer it over to the falcon tube. Sonicate to disperse the solids for efficient washing of the calcium hydroxide.
4. Repeat Step 3 two more times to ensure all calcium hydroxide is washed away.
5. Transfer the contents into a smaller centrifuge tube (1.5 mL), centrifuge at 15,500 rpm for 5 minutes and discard the water. Multiple washes of the larger falcon tube are recommended to prevent loss of any product.
6. After, water is discarded we are expected to have a wet solid. The final drying was performed in an oven at 80 °C overnight. An aluminium foil was used to cover the lid of the centrifuge tube. Several holes were made to allow water to leave.
7. Solid was removed from centrifuge tube and weighed

Ammonia was quantified using the indophenol method as described in previous work.[1]

Differential Scanning Calorimeter

Samples of ammonium formate were dried at 80 °C under an ammonia atmosphere for > 6 hours before being sealed by parafilm and stored in a dessicator until tested on the Differential Scanning Calorimeter (DSC). A TA Instruments DSC250 DSC was used to calculate the melting temperature and enthalpy of fusion for ammonium formate. The DSC was calibrated using a

sample of benzoic acid under the same conditions. The samples were placed in a sealed boat and then the temperature was ramped at a speed of 1 °C per minute from ambient condition to 140°C. Integration of the heat flux to calculate enthalpy was done using the trapezoidal method on the raw data for a given window centered on the heat flux peak (**Figure S5**). The exact window bounds had little impact on the integrated enthalpy or melting temperature (< 5% change in enthalpy and negligible change in temperature).

COSMO-RS Calculations

COSMO-RS is a program that can be used for calculating thermodynamic properties of (mixed) fluids.[2] It is a combination of COSMO with a statistical thermodynamics treatment of interacting surfaces making it more powerful.[3] It has been used successfully to model solute-solvent systems and shown to perform well for multi-phase solvents over temperature regions.[4,5] In this case, we have a ternary solvent of ammonium, formate, and water as ammonium formate is largely present in the dissociated form while water primarily exists as one molecule. We are interested in ammonia vapor pressures hence it is declared as the solute. For the species in this system, we were able to use COSMO-RS libraries. COSMO-RS outputs activity of ammonia in the solvent along with the Gibbs free energy of solvation. These values can be used to compute the equilibrium vapor pressure above the solvent using the equation shown below,

$$P_i = P_{tot}\gamma_i e^{\left(\frac{-\Delta G_{solv,i}}{RT}\right)}$$

where P_{tot} is the total pressure, γ_i is the activity coefficient of species i, $\Delta G_{solv,i}$ is the solvation free energy of species i, R is the gas constant, and T is the temperature.

Supporting Discussions

Comparison to Previous Literature

Table S1. Comparison with previous literature.

Paper	Electrolyte /system	Catalyst	Temperature	Experiments	Where we differ
Aldous 2010[6]	1:1.25 AF:FA	Pt	RT	CVs for half reactions; long-term tests; quantification of products	- Different electrodes<br>- Elevated temperature<br>- Stability at higher currents after 1 hr<br>- Thermodynamic discussion
Ohyama et al. 2007[7]	1 M AF, 1M FA both w/ 0.5 M H_2SO_4	Pt, PtIr	RT	CVs; 10 min CA	- Elevated temperature<br>- Longer stability<br>- Product/system analysis<br>- Thermodynamic discussion
Ohyama et al. 2008[8]	0.1 M AF	Pt, PtIr, PtRu	RT	CVs; 10 min CA	- Elevated temperature<br>- Longer stability<br>- Product/system analysis<br>- Thermodynamic discussion
Aoki et al. 2008[9]	0.1 M AF	Pt, PtPd	RT	CVs; 10 min CA	- Elevated temperature<br>- Longer stability<br>- Product/system analysis<br>- Thermodynamic

					discussion
Gao et al. 2012[10]	1M FA + 0.25-4M NaF	Pt	RT	CVs; 10 min CA	No AF
Guo et al. 2019[11]	< 10 M AF:FA in water	Au3Pd	52 – 92°C	Rate law determination, catalyst stability	No electrochemistry
Sridhar 2014[12]	AF	Au/TiO2	200-300°C	Product distribution; rate law determination; TOF calculation	No electrochemistry
Sridhar 2015[13]	AF	Au/TiO2	150-300°C	Selectivity and rate law determination; conversion as a function of catalyst support; TOF calculation	No electrochemistry, definitely produces CO
Zhou 2016[14]	<10 M AF-FA mixtures in water	Pd/C	30 – 50°C	Composition dependence; TOF calculation	No electrochemistry
Dobrovolna 2000[15]	AF in MeOH/water mixture	Pd/C	20°C	TOF calculation; product distribution	No electrochemistry
**This work**	**AF 90 w/w% with water**	**Pd, Au, Pt**	**105°C, RT, 80°C**	**Overpotential calculation, CVs, 1 hr long CAs, product distribution, thermodynamic + kinetic discussion**	

Note: "AF" indicates ammonium formate; "FA" indicates formic acid; "RT" indicates room temperature; "CV" indicates cyclic voltammagrams; "CA" indicates chromoapperometry.

Comparison to Thermochemical Decomposition

Table S2. Comparison of reaction rate with previous literature

Paper	Highest TOF (hr^{-1})	Temperature	Catalyst	Catalyst surface area (cm^3/g)	Equivalent current (mA/cm^2)
Guo et al. 2019[11]	407.5 hr^{-1}	92 °C	Au_3Pd_1/C	8.1E5	0.15
Sridhar 2015[13]	4.5 mol/g.hr[a]	300 °C	Au/La-TiO_2	1.1E6	0.23
Zhou 2016[14]	7959 hr^{-1}	50 °C	Pd/C	2.0E6	1.98
Dobrovolna 2000[15]	1.4 mol/g.hr[a]	50 °C	Pd/C	1.16E7	0.01
**This work**	**N/A**	**105 °C**	**Pd foil**	**N/A**	**>10**

[a] Converted from rate constants and reaction conditions

Here, we assume that all TOFs represent moles reacted per mole catalyst per hour (units converted from those given in the papers). Using the catalyst mass, known density of the materials (an assumption for nanoparticles), and the radius of the particles (we assume the particles are spheres), we can calculate the surface area of the catalysts and the equivalent current:

$$SA\ per\ mass = \frac{1}{density} * \frac{4\pi R^2}{\frac{4}{3}\pi R^3} = \frac{mass}{density} * \frac{3}{R}$$

$$i_{equivalent} =$$

$$TOF * \frac{1\ hr}{3600\ s} * \frac{1}{Molar\ mass\ catalyst} * \frac{1}{SA\ per\ mass} * \frac{2\ mol\ e^-}{1\ mol\ AF} * 96485 \frac{C}{mol\ e^-}$$

Influence of Temperature on Electrochemical Kinetics

Assume an anodic reaction obeys Marcus kinetics:

$$M \rightarrow M^{n+} + ne^- \quad (1)$$

$$i_a = nFk_a^0 c_M \exp\left(\frac{E_a}{RT}\right) = nFk_a^0 c_M \exp\left(\frac{-(\lambda_a - F\eta_a)^2}{4\lambda_a RT}\right) \quad (2)$$

where n is the number of electrons transferred (we will assume the first electron transfer is the RDS with $n-1$ transferred after the RDS), F is Faraday's constant, k_a^0 is the constant prefactor, λ_a is the reorganization energy, and $\eta_a = \phi_a - \phi_{a,eq} > 0$ is the overpotential relative to the equilbrium potential for the anodic half-reaction. R and T are the ideal gas constant and temperature, respectively, and c_M is the molar concentration of the reactant. We can quickly see that:

$$\log i_a = \log nFk_a^0 c_M - \frac{(\lambda_a - F\eta_a)^2}{4\lambda_a RT} \quad (3)$$

$$\frac{\partial \log i_a}{\partial \phi_a} = \frac{\partial \log i_a}{\partial \eta_a} \quad (4)$$

$$= -\frac{2(\lambda_a - F\eta_a)(-F)}{4\lambda_a RT} \quad (5)$$

$$= \frac{F}{2RT} - \frac{F^2\eta_a}{2\lambda RT} \quad (6)$$

We can see that both the expression for current as well as the Tafel slope ($\frac{\partial \log i_a}{\partial \phi_a}$) will approach the Butler-Volmer values in the limit $\eta << \lambda$. We will assume $\eta < \lambda$ for the rest of this derivation (low-overpotential regime).

We can then take some temperature derivatives:

$$\left.\frac{\partial \log i_a}{\partial T}\right|_\eta = \frac{(\lambda_a - F\eta_a)^2}{4\lambda_a RT^2} > 0 \quad (7)$$

So we see that in all cases, at constant overpotential, increasing temperature will lead to increasing currents.

But what happens to the overpotential in the case of increasing temperature?

$$-4\lambda RT\log\frac{i_a}{nFk_a^0c_M} = (\lambda_a - F\eta_a)^2 \tag{8}$$

$$-4\lambda R\log\frac{i_a}{nFk_a^0c_M}\partial T = 2(\lambda_a - F\eta_a)(-F)\partial\eta_a \tag{9}$$

$$\left.\frac{\partial\eta_a}{\partial T}\right|_{i_a} = \frac{-4\lambda_a R}{2(\lambda_a - F\eta_a)(-F)}\log\frac{i_a}{nFk_a^0c_M} \tag{10}$$

$$= \frac{2\lambda_a R}{F(\lambda_a - F\eta_a)}\log\frac{i_a}{nFk_a^0c_M} \tag{11}$$

$$= \frac{2\lambda_a R}{F(\lambda_a - F\eta_a)}\frac{-(\lambda_a - F\eta_a)^2}{4\lambda_a RT} \tag{12}$$

$$= \frac{-(\lambda_a - F\eta_a)}{2TF} > 0 \tag{13}$$

From the above, we see that *anodic* overpotentials decrease as temperature increases (by our original assumptions, $\lambda_a - F\eta_a > 0$). The cathodic version of the above is straightforward with a simple sign switch ($\eta_c < 0$):

$$N + ne^- \rightarrow N^{n-} \tag{14}$$

$$\log i_c = \log nFk_c^0c_N - \frac{(\lambda_c + F\eta_c)^2}{4\lambda_c RT} \tag{15}$$

$$\left.\frac{\partial\eta_c}{\partial T}\right|_{i_c} = \frac{(\lambda_c - F\eta_c)}{2TF} > 0 \tag{16}$$

Similarly, we see that *cathodic* overpotentials decrease with increasing temperature (remember that $\eta_c < 0$). We can then subtract the anodic derivative from the cathodic derivative to get an expression for the change in total cell potential with temperature (note that $i = i_c = i_a$):

$$\left.\frac{\partial\eta_c}{\partial T}\right|_{i_c} - \left.\frac{\partial\eta_a}{\partial T}\right|_{i_a} = \left.\frac{\partial\Delta\phi}{\partial T}\right|_i - \left.\frac{\partial\Delta\phi_{eq}}{\partial T}\right|_i$$

$$\left.\frac{\partial\Delta\phi}{\partial T}\right|_i = \frac{\partial\Delta\phi_{eq}}{\partial T} + \frac{(\lambda_c + F\eta_c)}{2TF} + \frac{(\lambda_a - F\eta_a)}{2TF}$$

$$= \frac{\partial\Delta\phi_{eq}}{\partial T} + \frac{(\lambda_c + \lambda_a + F\Delta\phi - F\Delta\phi_{eq})}{2TF}$$

(17)

(18)

(19)

We know that $-nF\Delta\phi_{eq} = \Delta G^0_{rxn} = \Delta H^0_{rxn} + -T\Delta S^0_{rxn}$. So,

$$\frac{\partial \Delta\phi_{eq}}{\partial T} = \Delta S^0_{rxn}/nF \tag{20}$$

$$\left.\frac{\partial \Delta\phi}{\partial T}\right|_i = \Delta S^0_{rxn}/nF + \frac{(\lambda_c + \lambda_a + F\Delta\phi - F\Delta\phi_{eq})}{2TF} \tag{21}$$

Let's **assume we have non-gas reactants turning into gaseous products**, $\Delta S^0_{rxn} > 0$. Therefore, all terms in the above equation are positive and we find:

$$\left.\frac{\partial \Delta\phi}{\partial T}\right|_i > 0 \tag{22}$$

For an electrolyzer, $\Delta\phi < 0$, so this means that the overall cell potential ($|\Delta\phi|$) required to drive a reaction at some constant current, *i*, will decrease as temperature increases. For a fuel cell, $\Delta\phi > 0$, so this means that the overall cell potential ($|\Delta\phi|$) we extract from a reaction at some constant current, *i*, will increase as temperature increases.

Supporting Analysis

Energy Density Calculations

As mentioned in the main text, there are multiple ways to calculate and compare the energy density of different molecules. One standard method for calculating energy density is to calculate the *higher heating value* (HHV), which is generally defined as the heat of combustion where the products are cooled to 25°C. The *lower heating value* (LHV) has multiple definitions, but generally does not include the latent heat of vaporization for water (e.g., the products of combustion are kept at 150°C). For the equilibrium potential, we care about the Gibbs free energy of the combustion reaction, i.e., the useful work that can be extracted from the reaction. We define combustion in this case as the reaction of one mole of fuel with stoichiometric oxygen to produce a combination of water, nitrogen gas, or carbon dioxide. The raw data and resulting energy densities on a volume basis are provided for select fuels (**Table S1**).

Table S1. Energy density thermodynamics for select fuels

**Compound**	ΔH_c **(kJ/mol)**[16]	ΔG_c **(kJ/mol)**	ρ **(kg/m³)**	**Mass (g/mol)**	**HHV (kWh/L)**	**Energy Density (kWh/L**	**Storage Conditions**
H_2 (gas)	-285.8	-237.1	39.2	2.0	1.5	1.3	RT, 700 bar
H_2 (liq)	-285.8	-237.1	71.0	2.0	2.8	2.3	20 K, 1 bar

NH_3 (liq)	-382.8	-339.3	602.9	17.0	3.8	3.3	RT, 10 bar
HCOOH (liq)	-254.3	-269.0	1220.0	46.0	1.9	2.0	RT, 1 bar
NH_4HCOO (s)	-540.6[17]	-570.9	1280.0	63.1	3.0	3.2	RT, 1 bar

Note that thermodynamic data for all of the above fuels is readily available except in the case of solid ammonium formate. The enthalpy of solid ammonium formate is tabulated[17] but the Gibbs free energy (or the absolute entropy) is not. To calculate the Gibbs free energy, we can use volume-based thermodynamics to calculate the absolute entropy,[18,19] then combine the entropy with the enthalpy to get the Gibbs free energy. The entropy of ionic solids is linearly proportional to the volume of the formula unit (equivalently the ratio of the molar mass and the density). Previous research on inorganic salts found the following relation:[18]

$$S_{298}^{0}\left(\frac{J}{K\,.mol}\right) = (2.258 \pm 0.093)\left(\frac{M}{\rho}\left(\frac{g}{cm^3}\right)\right) + (15 \pm 6)$$

They found that for a wide range of inorganic salts, this equation accurately predicted the entropy with an average unsigned percent error of 11.5%. We verified the applicability of this expression for ammonium formate by testing it with ammonium chloride and sodium formate, both of which have tabulated entropies (**Table S3**).[16]

Table S3. Calculation of absolute entropies for sample compounds and ammonium formate

	**Ammonium Chloride**	**Sodium Formate**	**Ammonium Formate**
Measured[16]	94.85	103.76	

$S^0_{298}(J.K^{-1}.mol^{-1})$			
Calculated[18] $S^0_{298}(J.K^{-1}.mol^{-1})$	93.9 ± 9.3	95.0 ± 9.3	126.2 ± 10.6
% Error	1%	8%	

As can be seen, the calculations accurately predict the absolute entropy of ionic solids to within 10% error.

Resistance of cell over time

As discussed in the main text (Figure 2), the solution resistance changes throughout the course of the chronopotentiometry hold (see Experimental Procedure above). This change is likely due to changing electrolyte composition over time, such as an increase in acidity at the anode. During the course of the one hour chronopotentiometry test, the solution resistance increases by ca. 3-5 Ω, regardless of anode material (**Figure S2**). At 10 mA applied current, this corresponds to an increased voltage of ca. 30-50 mV during the course of an hour, which matches with the observer slight increase in cell voltage (Figure 2).

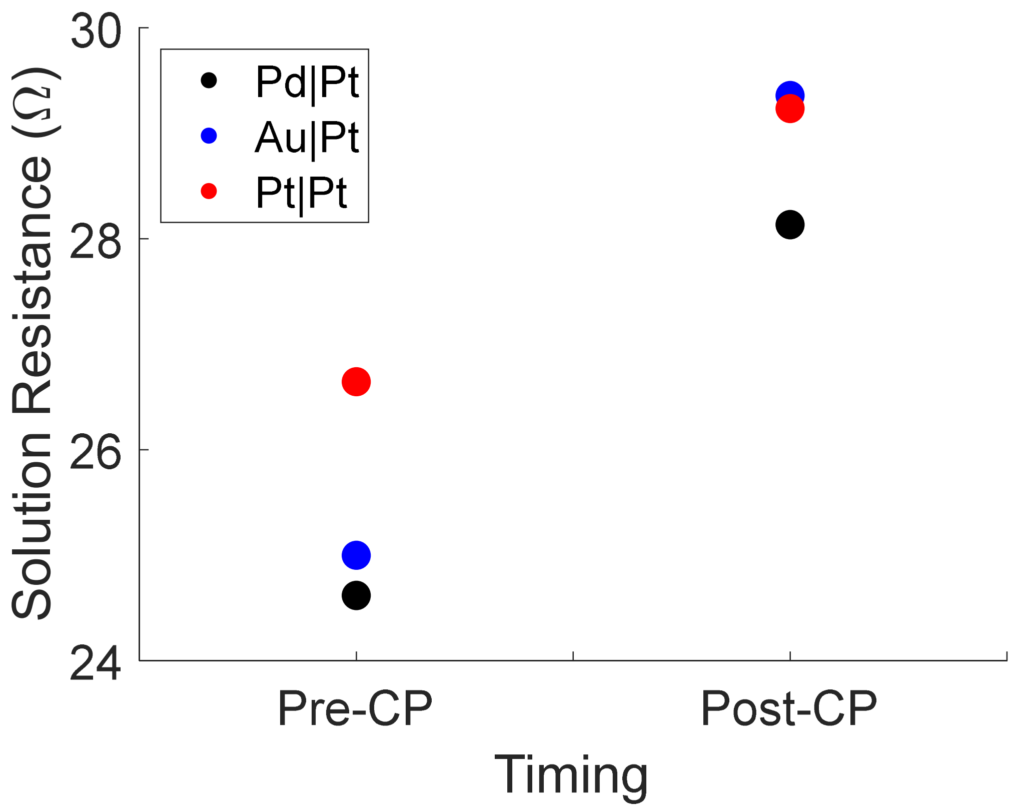

Figure S2. Solution resistance before and after the chronopotentiometry test. PEIS was done before and after the 60 minutes of 10 mA applied current, and the solution resistance was extracted from the PEIS by finding either where the imaginary part of the impedance crosses zero or the value of the impedance at a frequency of 10 kHz. Each point represents the average of at least three trials.

Hydrogen quantification

First, the GC was calibrated for hydrogen gas (**Figure S3**).

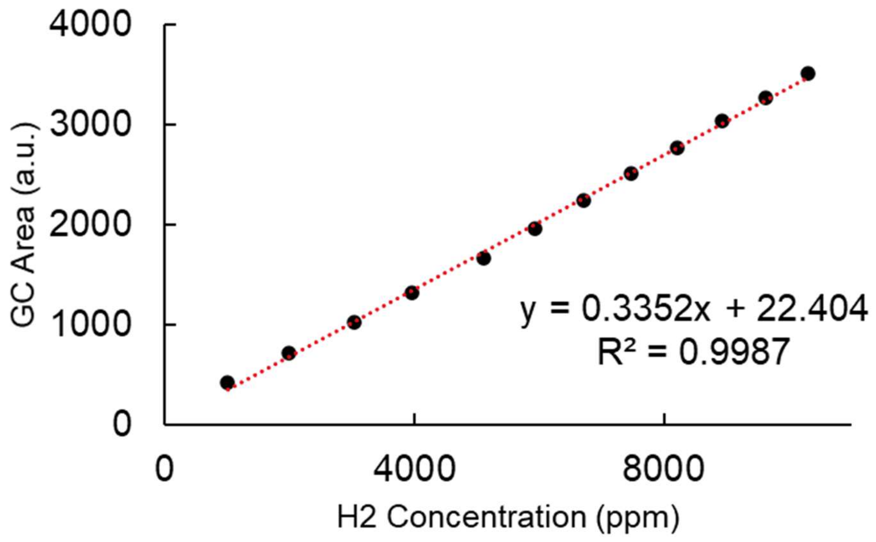

Figure S3. Calibration curve for hydrogen quantification on GC

The experimental setup is described above. Using a Pd anode and Pt cathode, the following Faradaic efficiency data was collected (**Figure S4**). The Faradaic efficiency reported in the main text (Figure 3) was an average of the data points collected after one hour had passed to allow for the system to reach steady state.

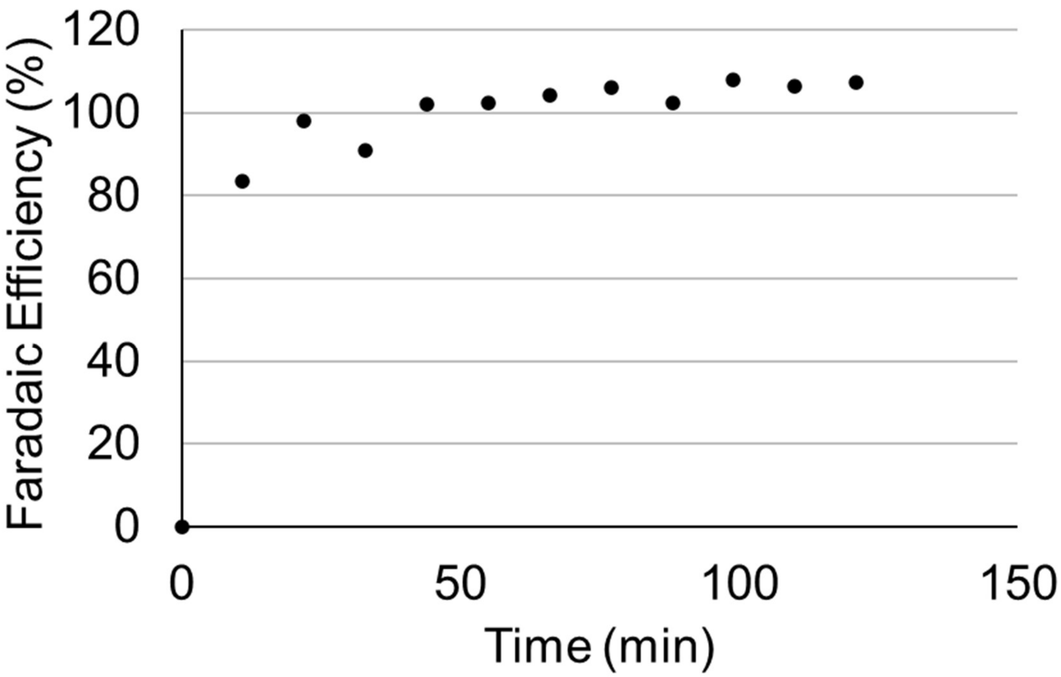

Figure S4. Faradaic efficiency toward hydrogen at the cathode over time. The system reached steady state after ~60 minutes due to the large residence time of the cell and the post-traps.

Carbon dioxide quantification

Carbon dioxide quantification was performed as described above. Two trials were taken with the following raw data results. These values were averaged and reported in the main text (Figure 3).

Table S4. Carbon dioxide quantification trials

	Charge (C)	Mass of $CaCO_3$ expected (mg)	$CaCO_3$ Recovered (mg)
Exp #1	25.56	13.2	13.1
Exp #2	25.56	13.2	12.9

Thermodynamic landscape

The thermodynamic landscape presented in the main text (Figure 4), was calculated as follows. Each step of the landscape was calculated relative to the final products, which are nitrogen, water, and carbon dioxide at 25°C. The "energy" at each step, is therefore simply the negative of the reaction enthalpy (or Gibbs free energy) of the reactants at each step reacting to form the products at the final step. The change is sign is for intuitive interpretation of the plot. To calculate the Gibb's free energy at 120°C, the Gibbs-Helmholtz equation was used with the enthalpy and Gibb's free energy at 25°C:

$$\frac{\Delta G(120^{\circ}C)}{393.15\ K} = \frac{\Delta G(25^{\circ}C)}{298.15\ K} + \Delta H\left(\frac{1}{393.15\ K} - \frac{1}{298.15\ K}\right)$$

Note that an assumption in this equation is that $\Delta H(25^{\circ}C) = \Delta H(120^{\circ}C) = \Delta H$ and that even for the Gibb's free energies at 120°C, the values are still *relative to the products at 25°C*, not the products at 120°C (the products at 120°C are, in fact, 38.5 kJ/mol less stable than the products

at 25°C). The raw values for the thermodynamic landscape (Figure 4 in main text) are provided (**Table S5**).

Table S5. Raw values for the thermodynamic landscape. All energies are in kJ/mol.

**ΔH**	**ΔG (25°C)**	**ΔG (120°C)**	**Step**
321	528	556	Ammonium Formate + 1.25O_2
364	539	556	Ammonium Formate (l) + 1.25O_2
449	534	522	NH3 + H2 + 1.25O2 + CO2
495	550	529	.5N2 + 2.5H2 + 1.25O2 + CO2
0	0	0	.5N2 + 2.5H2O (g) + CO2

Equilibrium potential calculations

Ammonium formate decomposition into carbon dioxide, hydrogen and nitrogen is a 5 electron process. Similarly, ammonium formate decomposition into carbon dioxide, hydrogen, and ammonia is a 2 electron process. Using the fact that $\mathrm{E^{eq}} = -\frac{\Delta \mathrm{G}}{\mathrm{nF}}$, we can calculate the equilibrium potential for various reactions used in the main text to calculate overptoentials (**Table S6**).

Table S6. Equilibrium potential calculations for various relevant reactions

	Temperature	ΔG_{rxn} (kJ/mol)	E^{eq}(mV)
$AF(s) \rightarrow \frac{5}{2}H_2 + CO_2 + \frac{1}{2}N_2$	25°C	21.9	-45
$AF(s) \rightarrow \frac{5}{2}H_2 + CO_2 + \frac{1}{2}N_2$	120°C	-26.6	55
$AF(\ell) \rightarrow \frac{5}{2}H_2 + CO_2 + \frac{1}{2}N_2$	25°C	11.5	-24
$AF(\ell) \rightarrow \frac{5}{2}H_2 + CO_2 + \frac{1}{2}N_2$	120°C	-26.6	55
$AF(s) \rightarrow H_2 + CO_2 + \frac{1}{2}NH_3$	25°C	5.5	-29
$AF(s) \rightarrow H_2 + CO_2 + \frac{1}{2}NH_3$	120°C	-33.5	174
$AF(\ell) \rightarrow H_2 + CO_2 + \frac{1}{2}NH_3$	25°C	-4.9	25
$AF(\ell) \rightarrow H_2 + CO_2 + \frac{1}{2}NH_3$	120°C	-33.5	174

Differential Scanning Calorimeter

The enthalpy and temperature of fusion for ammonium formate were calculated as described above. The DSC heat flux was calibrated using benzoic acid, which was known to have a melting temperature near that suspected of ammonium formate.

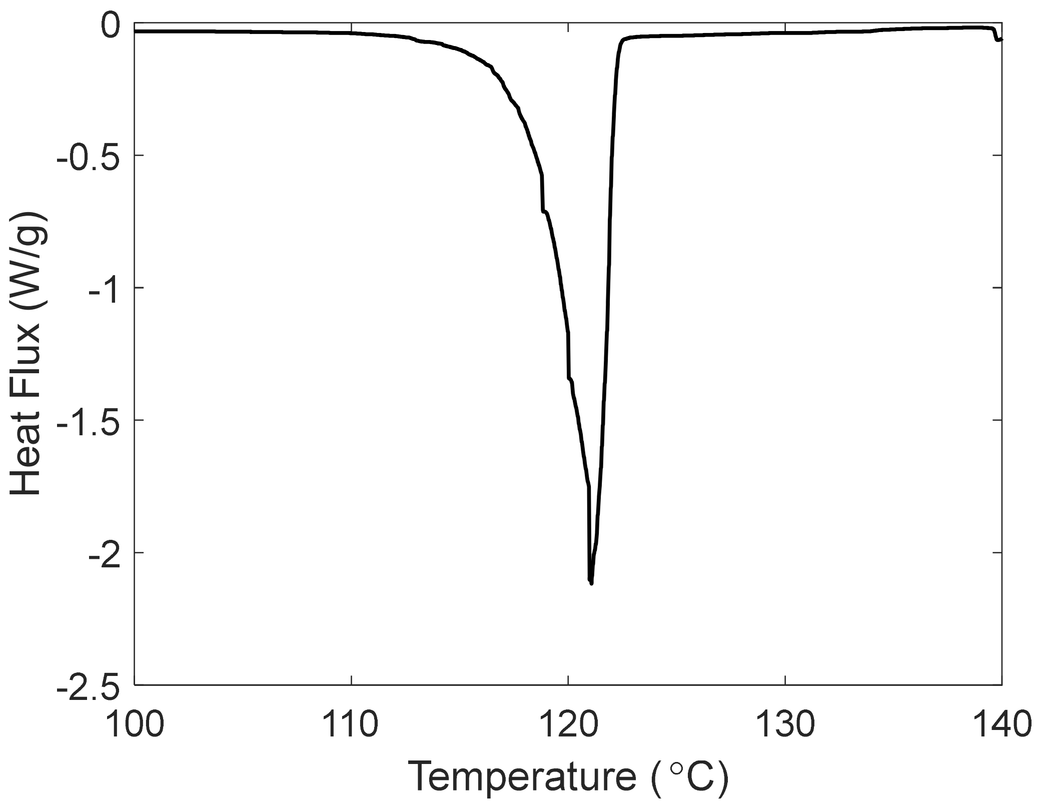

Figure S5. Example plot of DSC data for ammonium formate. The peak was integrated to find the enthalpy and the weighted average temperature was used to find the peak temperature.

Table S7. Raw data from DSC (T in °C and ΔH_{fus} in kJ/mol) .

Ammonium Formate						Benzoic Acid Standard			
Trial 1		Trial 2		Trial 3		Trial 1		Trial 2	
T	ΔH_{fus}	T	ΔH_{fus}	T	ΔH_{fus}	T	ΔH_{fus}	T	ΔH_{fus}
119.8	21.00	121.1	23.02	119.0	20.26	122.8	9.19	122.8	8.07

The average temperature of fusion for benzoic acid was measured to be 122.8°C and the ΔH_{fus} was measured to be 8.63 kJ/mol. The known melting point is 122.2°C and 17.4 kJ/mol.[16,20] We

can calibrate the enthalpy area by the ratio of the actual value and the measured value for benzoic acid. Thus, the true value ΔH_{fus} for ammonium formate is **43.2 kJ/mol**. We will assume that the temperature of the DSC is properly calibrated since the value is within noise of the benzoic acid standard. Thus, the melting temperature of ammonium formate is **120.0°C**.

Control Experiments

We performed control experiments at 105°C and 120°C. At 120°C, we found that 1.35E-3 moles of ammonia evolved from a pure ammonium formate electrolyte over the course of 100 minutes (stating amount 0.19 moles of ammonium formate). There were also trace amounts of formic acid in the post-trap (detected via NMR) and trace amounts of carbon monoxide and carbon dioxide detected via in-line gas chromatograph analysis. At 105°C, we found 7.5E-4 moles of ammonia evolved in the 90 w/w% ammonium formate electrolyte after 25 minutes (stating amount 0.17 moles of ammonium formate). There was no measurable formic acid in the post-trap at 105°C and no carbon monoxide or carbon dioxide were detected via gas chromatograph. Overall, we can conclude that ammonia will slowly evaporate over time in an open system, but that at 105°C, the formate is thermally stable. At 120°C, the formate is not thermally stable, and trace amounts of formic acid will also evaporate.

Temperature Dependence Referenced Experiments

Experiments in a three-electrode setup were performed at the regular conditions (105°C, 90 w/w% ammonium formate and 10 w/w% water), as well as at room temperature (saturated solution of ammonium formate in water, assumed to be approximately 22°C) and at 80°C with

75 w/w% ammonium formate and 25 w/w% water (chosen to be close, but under the saturation point at that temperature). In all cases, the same experimental setup as previously described was used (Pd anode and Pt cathode) with the addition of a Pt wire as a pseudo-reference. In addition to anodic overpotentials versus a Pt pseudo-reference, the systems at 22°C and 80°C were calibrated with a ferricyanide redox couple so that the results could be plotted vs SHE and vs the formate oxidation potential (Table S8). Unfortunately, ferricyanide is not stable at 105°C, so exact overpotentials vs the equilibrium potential could not be determined at that temperature.

Table S8. Calibration for ferricyanide redox couple as a function of temperature

Temperature	22°C	80°C	105°C
Experimental Ferricyanide redox (V vs Pt pseudo-reference)	0.475	0.432	N/A
Literature Ferricyanide redox (V vs SHE)[21]	0.4378	0.2804	0.2047
Calculated Formate oxidation (V vs SHE)[16,22]	-0.227	-0.203	-0.195
Calculated Pt pseudo-reference (V vs SHE)	-0.0445	-0.1512	N/A
Calculated Pt pseudo-reference (V vs Formate Oxidation)	0.19	0.0514	N/A

Temperature Dependence on System Equilibrium Composition

This system is a unique combination of water, ammonia, and formic acid. As a function of temperature, we expect to see water in liquid and gaseous form, formic acid in liquid, gaseous

and anion form, and ammonia as a gas and a cation. Accordingly, we can generate a variety of phase diagrams of the ammonium formate condensed phase (Figure S6) and the vapor phase (Figure S7).

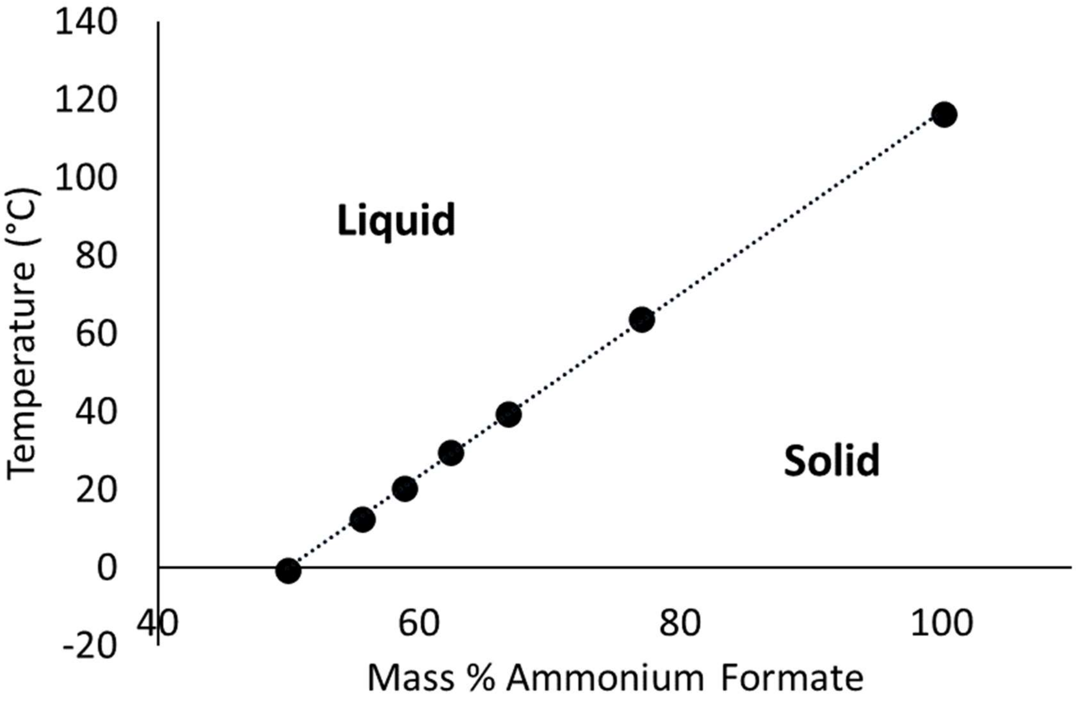

Figure S6. Phase diagram of condensed phases of ammonium formate-water system. Data taken from NIST.[23]

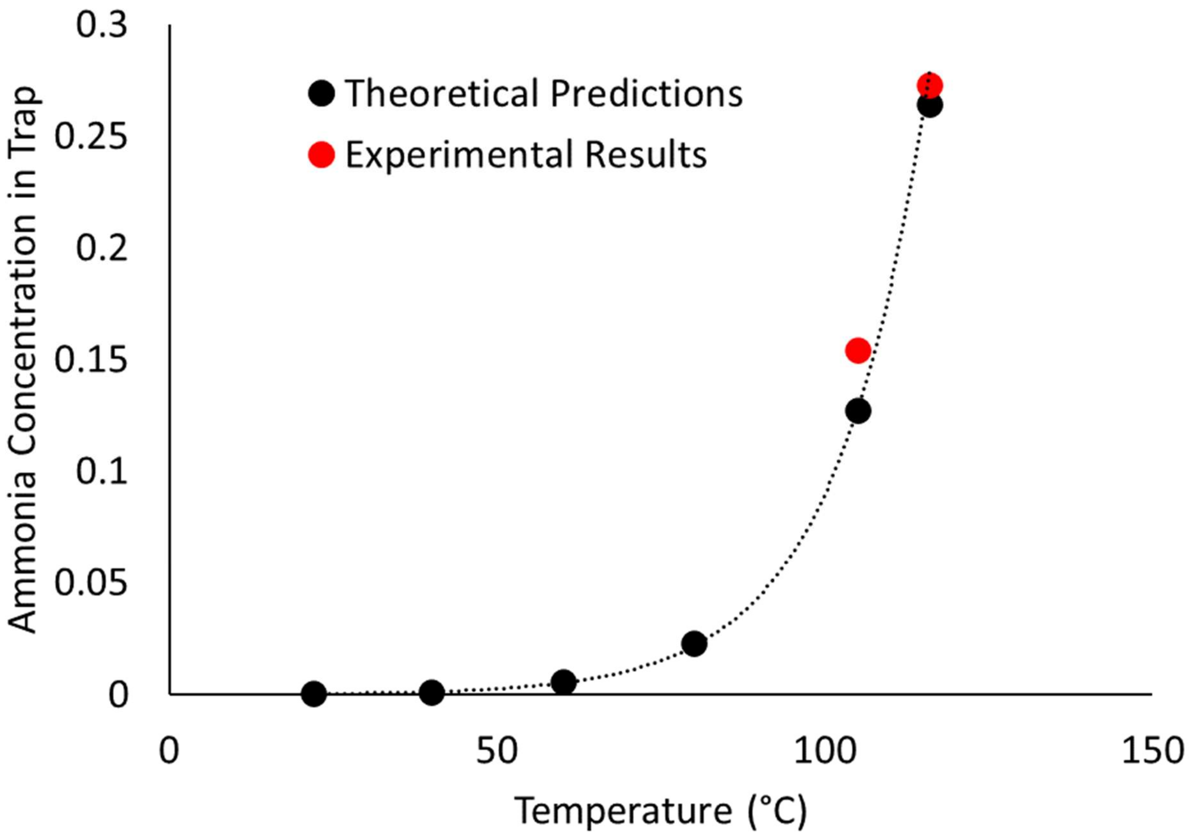

Figure S7. Expected ammonia evaporated by system and caught in trap based on carrier gas flow (10 SCCM per compartment) and experimentally observed ammonia in trap. Theoretical ammonia concentration calculated using the partial pressure from COSMO-RS calculations.

Supporting references

(1) Lazouski, N.; Schiffer, Z. J.; Williams, K.; Manthiram, K. Understanding Continuous Lithium-Mediated Electrochemical Nitrogen Reduction. *Joule* **2019**, *3* (4), 1127–1139. https://doi.org/10.1016/j.joule.2019.02.003.

(2) Pye, C. C.; Ziegler, T.; van Lenthe, E.; Louwen, J. N. An Implementation of the Conductor-like Screening Model of Solvation within the Amsterdam Density Functional Package — Part II.

COSMO for Real Solvents. *Canadian Journal of Chemistry* **2009**, *87* (7), 790–797. https://doi.org/10.1139/V09-008.

(3) Louwen, J. N.; Pye, C. C.; van Lenthe, E.; Austin, N.; McGarrity, E. S.; Xiong, R.; Sandler, S. I.; Burnett, R. I. AMS2018 COSMO-RS, SCM, Theoretical Chemistry. Amsterdam.

(4) Chung, Y.; Gillis, R. J.; Green, W. H. Temperature-dependent Vapor–Liquid Equilibria and Solvation Free Energy Estimation from Minimal Data. *AIChE Journal* **2020**, *66* (6). https://doi.org/10.1002/aic.16976.

(5) Chung, Y.; Vermeire, F. H.; Wu, H.; Walker, P. J.; Abraham, M. H.; Green, W. H. Group Contribution and Machine Learning Approaches to Predict Abraham Solute Parameters, Solvation Free Energy, and Solvation Enthalpy. *Journal of Chemical Information and Modeling* **2022**, *62* (3), 433–446. https://doi.org/10.1021/acs.jcim.1c01103.

(6) Aldous, L.; Compton, R. G. Clean , Efficient Electrolysis of Formic Acid via Formation of Eutectic , Ionic Mixtures with Ammonium Formate. *Energy & Environmental Science* **2010**, *3*, 1587–1592.

(7) Ohyama, K.; Kimura, C.; Aoki, H.; Kuwabata, S.; Sugino, T. Evaluation of Solid Ammonium Formate Oxidation for Polymer Electrolyte Fuel Cells. *ECS Transactions* **2007**, *11* (1), 1473–1477.

(8) Ohyama, K.; Sugino, T.; Nitta, T.; Kimura, C.; Aoki, H. Improvement of Anode Oxidation Reaction of a Fuel Cell Using Ammonium Formate with Pt-Ir Catalysts. *IEEJ Transactions EIS* **2008**, *128*, 1600–1604.

(9) Aoki, H.; Nitta, T.; Kuwabata, S.; Kimura, C.; Sugino, T. Improvement of Anodic Oxidation Reaction of a Fuel Cell Using Ammonium Formate with Pt-Pd Catalysts. *ECS Transactions* **2008**, *16* (2), 849–853.

(10) Gao, Y.; Tan, C.; Ye-ping, L. I.; Guo, J.; Zhang, S. Formic Acid-Formate Blended Solution : A New Fuel System with High Oxidation Activity. *International Journal of Hydrogen Energy* **2011**, *37* (4), 3433–3437. https://doi.org/10.1016/j.ijhydene.2011.11.077.

(11) Guo, X. T.; Zhang, J.; Chi, J. C.; Li, Z. H.; Liu, Y. C.; Liu, X. R.; Zhang, S. Y. Efficient Dehydrogenation of a Formic Acid-Ammonium Formate Mixture over Au 3 Pd 1 Catalyst. *RSC Advances* **2019**, *9* (11), 5995–6002. https://doi.org/10.1039/c8ra09534e.

(12) Sridhar, M.; Peitz, D.; van Bokhoven, J. A.; Kröcher, O. Ammonium Formate Decomposition over Au/TiO2: A Unique Case of Preferential Selectivity against NH3 Oxidation. *Chemical Communications* **2014**, *50* (53), 6998–7000. https://doi.org/10.1039/c4cc00196f.

(13) Sridhar, M.; Ferri, D.; Elsener, M.; van Bokhoven, J. A.; Kröcher, O. Promotion of Ammonium Formate and Formic Acid Decomposition over Au/TiO2 by Support Basicity under SCR-Relevant Conditions. *ACS Catalysis* **2015**, *5* (8), 4772–4782. https://doi.org/10.1021/acscatal.5b01057.

(14) Zhou, J. P.; Zhang, J.; Dai, X. H.; Wang, X.; Zhang, S. Y. Formic Acid–Ammonium Formate Mixture: A New System with Extremely High Dehydrogenation Activity and Capacity. *International Journal of Hydrogen Energy* **2016**, *41* (47), 22059–22066. https://doi.org/10.1016/j.ijhydene.2016.10.015.

(15) Dobrovolna, Z.; Cerveny, L. Ammonium Formate Decomposition Using Palladium Catalyst. *Research on Chemical Indermediates* **2000**, *26* (5), 489–497.

(16) Burgess, D. R. Thermochemical Data. In *NIST Chemistry WebBook, NIST Standard Reference Database Number 69*; Linstrom, P. J., Mallard, W. G., Eds.; National Institute of Standards and Technology: Gaithersburg, MD. https://doi.org/10.18434/T4D303.

(17) *Thermodynamic and Transport Properties of Organic Salts*; Franzosini, P., Sanesi, M., Eds.; 1980.

(18) Jenkins, H. D. B.; Glasser, L. Standard Absolute Entropy, S298o, Values from Volume or Density. 1. Inorganic Materials. *Inorganic Chemistry* **2003**, *42* (26), 8702–8708. https://doi.org/10.1021/ic030219p.

(19) Glasser, L.; Jenkins, H. D. B. Predictive Thermodynamics for Ionic Solids and Liquids. *Physical Chemistry Chemical Physics* **2016**, *18* (31), 21226–21240. https://doi.org/10.1039/c6cp00235h.

(20) James S. Chickos; William E. Acree, Jr.; Joel F. Liebman; Students of Chem 202 (Introduction to the Literature of Chemistry), U. of M.--St. L. Heat of Fusion Data. *NIST Chemistry WebBook, NIST Standard Reference Database Number 69*. National Institute of Standards and Technology. https://doi.org/10.18434/T4D303.

(21) Nordstrom, D. K. *Thermochemical Redox Equilibria of &Bell's Solution*; Pergamon Prffs, 1977; Vol. 41.

(22) Oelkers, E. H.; Helgeson, H. C.; Shock, E. L.; Sverjensky, D. A.; Johnson, J. W.; Pokrovskii, V. A. Summary of the Apparent Standard Partial Molal Gibbs Free Energies of Formation of Aqueous Species, Minerals, and Gases at Pressures 1 to 5000 Bars and Temperatures 25 to 1000 °C. *Journal of Physical and Chemical Reference Data*. 1995, pp 1401–1560. https://doi.org/10.1063/1.555976.

(23) Balarew, C.; Dirkse, T. P.; Golubchikov, O. A.; Salomon, M.; Trendafilova, S.; Tepavitcharova, S.; Ageyeva, T.; Baldini, P.; D'Andrea, G. IUPAC-NIST Solubility Data Series. 73. Metal and Ammonium Formate Systems Volume Editors. *Journal of Physical and Chemical Reference Data* **2001**, *30* (1), 1–8. https://doi.org/10.1063/1.1354207.
